# Automated post-run analysis of arrayed quantitative PCR amplification curves using machine learning

**DOI:** 10.12688/gatesopenres.16313.1

**Published:** 2025-01-20

**Authors:** Ben J. Brintz, Darwin J. Operario, David Garrett Brown, Shanrui Wu, Lan Wang, Eric R. Houpt, Daniel T. Leung, Jie Liu, James A. Platts-Mills

**Affiliations:** 1 University of Utah Department of Internal Medicine, Salt Lake City, Utah, USA; 2 University of Virginia, Charlottesville, Virginia, USA; 3 Qingdao University School of Public Healh, Qingdao, Shandong, China

**Keywords:** qPCR, PCR amplification, cycle threshold, machine learning

## Abstract

**Background:**

The TaqMan Array Card (TAC) is an arrayed, high-throughput qPCR platform that can simultaneously detect multiple targets in a single reaction. However, the manual post-run analysis of TAC data is time consuming and subject to interpretation. We sought to automate the post-run analysis of TAC data using machine learning models.

**Methods:**

We used 165,214 qPCR amplification curves from two studies to train and test two eXtreme Gradient Boosting (XGBoost) models. Previous manual analyses of the amplification curves by experts in qPCR analysis were used as the gold standard. First, a classification model predicted whether amplification occurred or not, and if so, a second model predicted the cycle threshold (Ct) value. We used 5-fold cross-validation to tune the models and assessed performance using accuracy, sensitivity, specificity, positive predictive value (PPV), negative predictive value (NPV), and mean absolute error (MAE). For external validation, we used 1,472 reactions previously analyzed by 17 laboratory scientists as part of an external quality assessment for a multisite study.

**Results:**

In internal validation, the classification model achieved an accuracy of 0.996, sensitivity of 0.997, specificity of 0.993, PPV of 0.998, and NPV of 0.991. The Ct prediction model achieved a MAE of 0.590. In external validation, the automated analysis achieved an accuracy of 0.997 and a MAE of 0.611, and the automated analysis was more accurate than manual analyses by 14 of the 17 laboratory scientists.

**Conclusions:**

We automated the post-run analysis of highly-arrayed qPCR data using machine learning models with high accuracy in comparison to a manual gold standard. This approach has the potential to save time and improve reproducibility in laboratories using the TAC platform and other high-throughput qPCR approaches.

## Introduction

Quantitative PCR has become an important approach for detection of pathogens across etiologic studies of common infectious diseases syndromes, including diarrhea, pneumonia, and acute febrile illness, sepsis, and meningitis. ^1^^–^^5^ Because of the wide range of pathogens that can be implicated for each of these syndromes, platforms that provide simultaneous detection of a number of pathogens are preferred, and the use of quantitative PCR has helped differentiate etiologic detections from background carriage ^6^^,^^7^ as well as to interpret drug resistance and pathogen type from the stool metagenome. ^8^^,^^9^ The TaqMan Array Card (TAC) is one such platform and has the advantage of being a closed system with arrayed singleplex or duplex target detection of both DNA and RNA targets, as long as the assays can be adapted to universal cycling conditions. ^6^

A limitation of the use of these array cards is the need for manual post-run adjustment of target baselines. While the software that accompanies the instrument can perform an automated analysis of baselines and returns a cycle threshold (Ct) for each amplification curve, these are subject to error and thus require manual inspection and correction. Because each sample lane can include duplex qPCR assays in each of 48 sub-wells, with eight samples per card, there can theoretically be up to 768 individual PCR amplifications per card. Large studies may include thousands of samples and thus in excess of a million PCR amplifications to analyze manually. Further, while a protocol is used to guide the post-run analysis, it is intrinsically subject to interpretation and can result in variability in Ct calls between individual analysts.

A fully automated alternative to manual post-run analysis would save time and effort and improve reproducibility. We thus sought to use machine learning approaches to derive models for prediction of both PCR amplification and, when amplification was observed, Ct values using TAC data from two previously-published enteric infectious diseases studies. ^10^^,^^11^ We then performed out-of-sample validation and compared the prediction model to post-run analyses performed by laboratories as part of an external quality assessment (EQA). ^10^

## Methods

### Data

For model development and internal validation, we used amplification data from 165,214 quantitative real-time PCR (qPCR) reactions, all performed on the TAC platform for 40 reaction cycles. Manual amplification and Ct value calls were used as the gold standard and were performed by two experts in qPCR curve analysis at the University of Virginia (DJO, JL). These reactions amplified a wide range of enteric pathogen targets. Of these, 56,539 reactions were selected from a multisite cohort studying early-life enteric infections (MAL-ED), ^10^ and an additional 108,675 reactions from a study of the etiology of diarrhea in children participating in a rotavirus vaccine clinical trial in Niger. ^11^ Between the two combined data sources, 115,965 (70.2%) were measured using the FAM dye and 49,249 (29.8%) using the VIC dye. 20,828 (12.6%) reactions were duplexed, with amplification data for both FAM and VIC dyes against two separate targets. We standardized the fluorescence values by dividing the raw FAM and VIC values by the values from the ROX reference dye for the corresponding cycle, and then normalized the value such that for each of cycle, $F_{\mathrm{norm}}=F_{\text{stand}}-\left(F_{\min}-1\right)$ where $F_{\text{stand}}$ is the standardized fluorescence value for that cycle and $F_{\min}$ is the minimum standardized fluorescence value across all 40 cycles.

We used 132,171 (80%) of the combined 165,214 reactions as the training set and the remaining 33,043 (20%) as the internal validation testing set. For external validation and to compare the model predictions to real-world manual post-run analyses, we used 1,472 reactions from three TAC run files each analyzed by 17 scientists from 8 laboratories participating in an EQA as part of the MAL-ED study. ^10^ As a final comparator set, we compared to the “Relative Threshold” Ct calls made Thermo Fisher’s QuantStudio software using a proprietary algorithm. ^12^

### Modeling development

We fit two tree-based models using the eXtreme Gradient Boosting (XGBoost) algorithm, with only the normalized fluorescence values from each of the 40 cycles as inputs. The first model was used to classify whether target amplification occurred, and the second was used in the subset of reactions where amplification was observed to predict the Ct value. We tuned the models on the 80% training set using 5-fold cross-validation. We considered various values for the XGBoost hyperparameters ( Table 1). More details on the hyperparameter tuning can be found at https://xgboost.readthedocs.io/en/latest/parameter.html. We used the optimal hyperparameters to train the models on the 80% training set and applied them to both the 20% testing set and the 3 EQA files. A model prediction value greater than 0.5 was considered consistent with amplification. The second XGBoost model was used conditionally on the first model and thus trained only on reactions that were considered as true amplification by the gold standard.

**Table 1.** XGBoost Model tuning parameter values considered and optimal values for each model from 5-fold cross-validation on the training set.

Parameter	Values	Amplification model	Cycle classification model
eta	0.1, 0.3, 0.5, 0.7	0.5	0.1
max_depth	10-18	13	16
min_child_weight	1, 5, 10	1	10
subsample	0.5, 0.7, 1	0.7	1
gamma	0, 0.1, 0.2	0.1	0.2
colsample_bytree	0.5, 0.7, 1	0.7	1

### Modeling performance

For the classification model, we assessed performance in both internal and external validation using accuracy as well as other performance metrics calculated from the confusion matrix including sensitivity (Se), specificity (Sp), positive predictive value (PPV), and negative predictive value (NPV). For the Ct prediction, we assessed performance using mean absolute error (MAE), i.e., the average absolute distance between our prediction and the ground truth Ct value. For curves that we incorrectly classified as having amplified, we used a Ct of 40 as the gold standard value in the MAE calculation.

### Model prediction flags

Because the XGBoost model returned a continuous value that was then dichotomized for the prediction of amplification, we explored whether the absolute prediction value could be used to flag amplification curves for which the model performance was lower. Specifically, we returned a flag if the model probability was between 0.1 and 0.9 and then evaluated whether the flag was associated with disagreement between the gold standard and the model.

## Results

**Figure 1.**
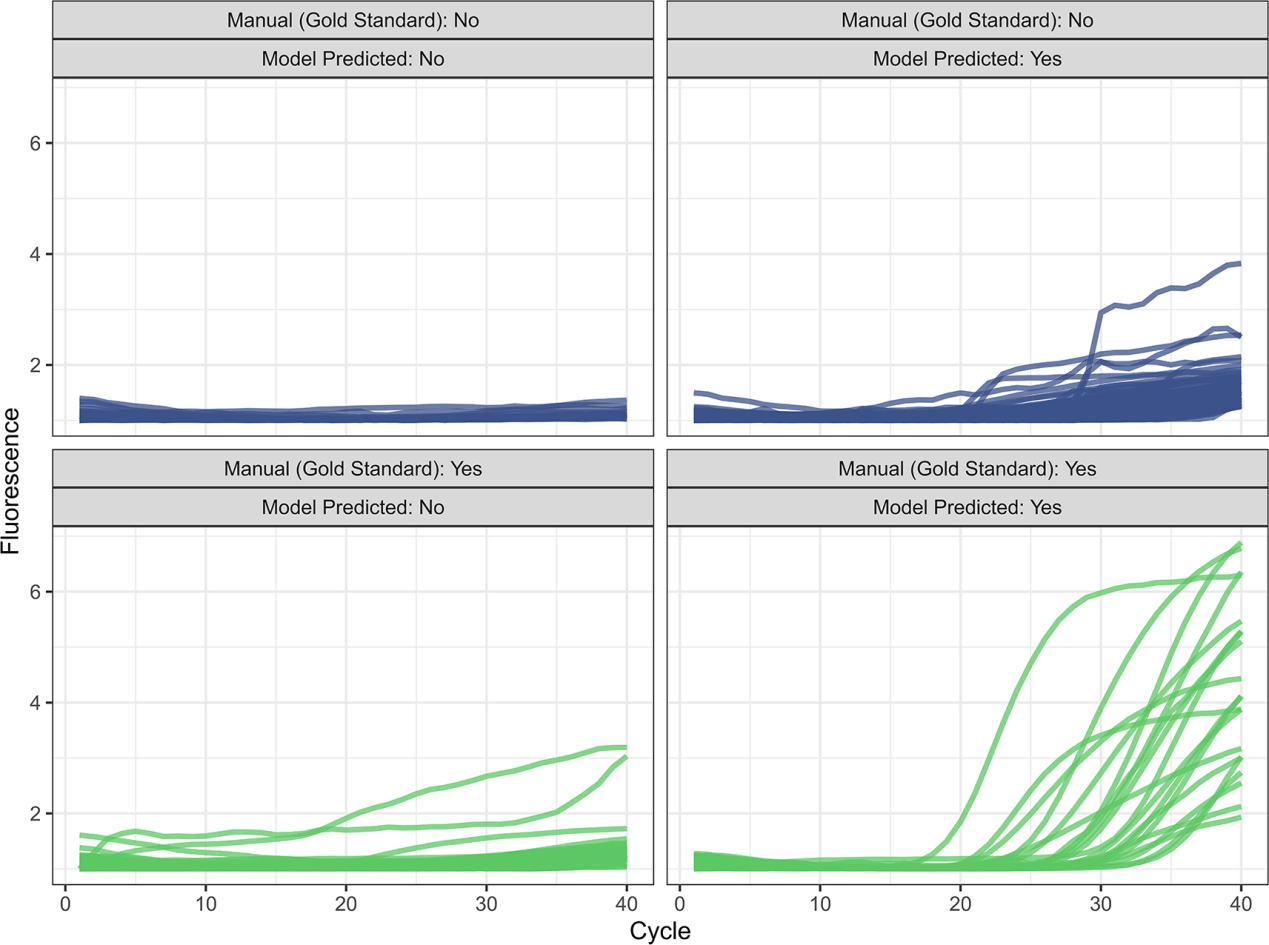
Plots of curves that were misclassified (off-diagonal) and 20 randomly-selected correctly classified curves (main-diagonal), with the cycle on the x-axis and normalized fluorescence on the y-axis in internal validation.

**Figure 2.**
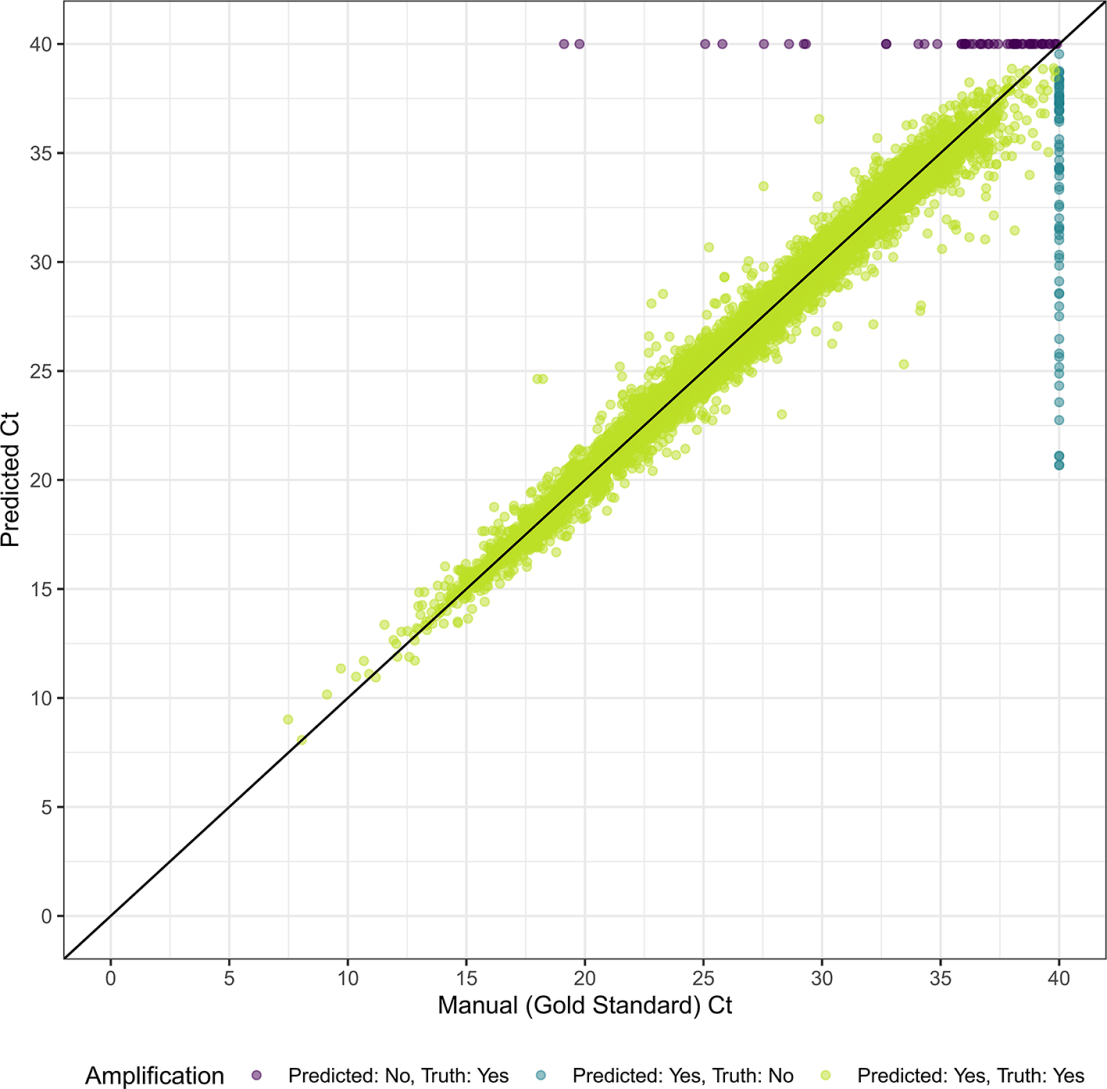
Manual (gold standard) Ct vs. automated (model-predicted) Ct in internal validation.

In 5-fold cross-validation, we first identified the optimal hyperparameter values for the two models ( Table 1). In internal validation on the 20% testing set, we achieve an accuracy of 0.996, a sensitivity of 0.997, a specificity of 0.993, a PPV of 0.998, and NPV of 0.991 ( Table 2). Of 33,043 reaction in the testing set, 125 (0.38%) were misclassified compared to the manual gold standard ( Figure 1). Our model misclassified 53 curves as not amplified, most of which had exponential growth at high cycle counts (mean=35.7, SD=4.86, 76% with a ground truth Ct ≥ 35). For the 72 curves our model misclassified as amplified, the mean predicted Ct was 33.2 (SD=5.33). We achieved an MAE of 0.590 when predicting the Ct value on the internal testing set. Excluding the reactions that the model incorrectly classified as true amplifications, the MAE reduced to 0.544 ( Figure 2). Model performance was similar between dyes and for singleplex vs. duplex reactions. Of the 125 misclassified reactions, 81 (64.8%) were flagged based on a continuous model prediction value between 0.1 and 0.9, in comparison to 124 (3.8%) of 32918 correctly classified reactions. The model also outperformed the automated relative threshold included in the machine software, which had an accuracy of 99.2% and a MAE of 1.00.

**Table 2.** Comparison of automated (model-based) and manual (gold standard) calls of amplification in internal and external validation.

			*Model call*	
			No amplification	Amplification
**Internal validation**	*Manual call*	No amplification	25166	72
		Amplification	53	7752
**External validation**	*Manual call*	No amplification	1118	2
		Amplification	3	349

**Figure 3.**
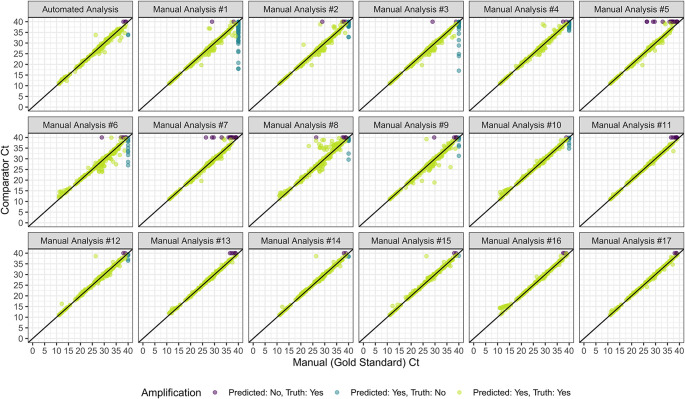
qPCR Ct predicted by either the automated (model-based) analysis or manual analyses performed by laboratory scientists as part of an EQA (Y axis), all shown in comparison to the manual gold standard (X axis). Manual analysis plots are ordered by site accuracy from lowest to highest.

**Figure 4.**
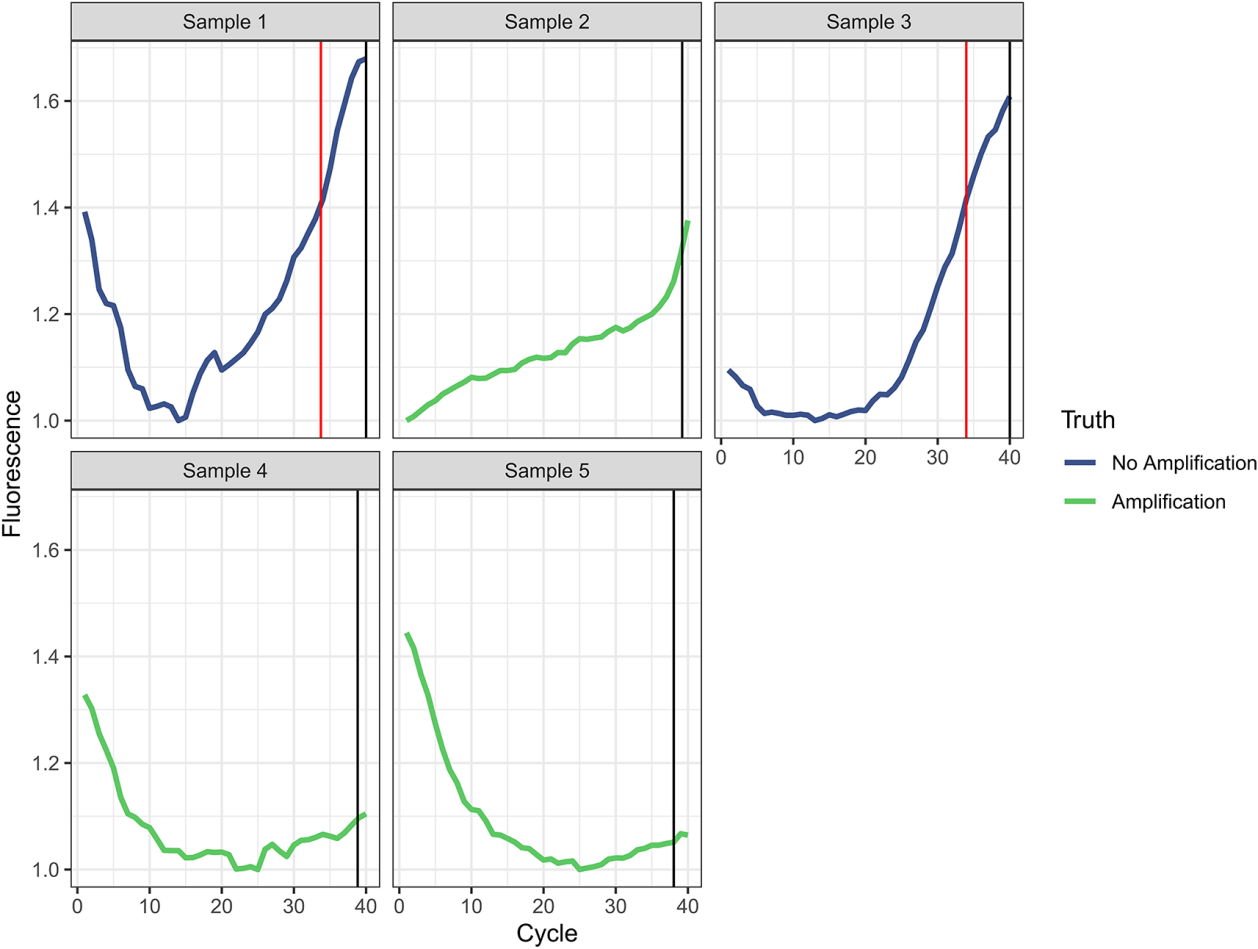
Amplification curves for incorrectly classified reactions in the external validation set. The vertical red line indicates the Ct predicted by the automated analysis and the vertical black line indicates the Ct assigned by the manual gold standard. In either case, a value of 40 was assigned if no amplification was identified.

In external validation, we achieved an accuracy of 0.997, a sensitivity of 0.998, a specificity of 0.991, a PPV of 0.997, an NPV of 0.994, and a MAE of 0.611 when classifying amplification and Ct values ( Table 2 and Figure 3). The MAE reduced to 0.579 when we excluded misclassified targets. Five of 1472 curves (0.34%) were incorrectly classified by the automated analysis compared to the manual gold standard ( Figure 4). For the three curves identified as true amplification by the manual gold standard but which the automated analysis missed, the Ct values approached 40 (mean=38.7, SD=0.59). The automated analysis had an accuracy for reaction classification better than 14 of 17 laboratory scientists ( Table 3). With a MAE for Ct prediction of 0.611, the automated analysis outperformed four of the 17 labs.

**Table 3.** Comparison of accuracy and mean absolute error (MAE) between each of the 17 laboratory scientists and the automated analysis.

User ID	Accuracy	MAE	MAE (Misclassified removed)
Manual Analysis #1	0.957	1.046	0.445
Manual Analysis #2	0.980	0.524	0.449
Manual Analysis #3	0.983	0.614	0.373
Manual Analysis #4	0.984	0.415	0.344
Manual Analysis #5	0.988	0.317	0.317
Manual Analysis #6	0.988	0.777	0.630
Manual Analysis #7	0.989	0.296	0.296
Manual Analysis #8	0.990	0.832	0.782
Manual Analysis #9	0.992	0.543	0.501
Manual Analysis #10	0.994	0.307	0.273
Manual Analysis #11	0.995	0.153	0.153
Manual Analysis #12	0.995	0.354	0.334
Manual Analysis #13	0.997	0.208	0.208
Manual Analysis #14	0.997	0.267	0.260
Automated Analysis	0.997	0.611	0.579
Manual Analysis #15	0.998	0.313	0.311
Manual Analysis #16	0.998	0.272	0.272
Manual Analysis #17	0.998	0.187	0.187

## Discussion

Using a large sample of qPCRs that were previously assessed using the background threshold method, we were able to accurately predict both presence of amplification and the qPCR Ct directly from normalized fluorescence values. In external validation, the accuracy was superb and as good as the best-performing laboratory scientists in a multisite EQA analysis. It also outperformed the automated algorithms included in the commercial qPCR software. This approach will allow for a substantial reduction in hands-on laboratory time, laboratory training and EQA, and provide an automated, fully reproducible method for the post-run analysis of arrayed qPCR.

Previous approaches to automating qPCR amplification and cycle threshold assessment have focused on first characterizing the curves by extracting the structure and key features, for example presence of a hook effect. ^13^^,^^14^ However, this requires additional computation and abstraction of the fluorescence data. Our approach minimizes data manipulation and is computationally efficient. We chose the tree-based algorithm, XGBoost, due to its ability to recognize complex interactions between predictors and nonlinear relationships between the predictors and outcomes, in addition to its efficiency. ^15^ All of the data used in this analysis came from qPCR on the stool metagenome. This is a convenient source of data for training and validating these models because of the high rate of target amplification (about one quarter of all qPCR reactions) and because of the wide range in pathogen quantity present. However, we would expect that these models would work equally well on qPCR reactions derived from other sample types. While the accuracy of amplification prediction in external validation was excellent, the cycle threshold error was higher than most of the labs, however the absolute difference in error was very small. We believe that this is a more than acceptable trade-off given the other clear advantages to this approach.

This automated post-run pipeline will be of particular value for multisite studies, removing an important source of between-site variation, as well as for studies where quick turnaround of qPCR data is valuable such as for clinical decision-making. The prediction models can be operationalized e.g. with a web portal, allowing for full-automated and reproducible amplification prediction, cycle threshold calling, data cleaning, and data aggregation. As with most evaluations of diagnostics, the absence of a true gold standard is a limitation, however in this case the goal was to consistently reproduce data generated using the best-available manual approach. In summary, we were able to accurately predict qPCR amplification and prediction by training machine learning models directly on normalized fluorescence data, which will allow for a substantial improvement in the efficiency and transparency of data generation.

## Data Availability

The latest version of the data and analysis code are available at https://github.com/bbrintz/qPCR-amp-call and are archived at Zenodo: https://zenodo.org/doi/10.5281/zenodo.13798820. Data are available under the terms of the Creative Commons Zero “No rights reserved” data waiver (CC0 1.0 Public domain dedication).

## References

[1] Cohen AL Platts-Mills JA Nakamura T : Aetiology and incidence of diarrhoea requiring hospitalisation in children under 5 years of age in 28 low-income and middle-income countries: Findings from the global pediatric diarrhea surveillance network. *BMJ Glob. Health.* 2022;7:e009548. 10.1136/bmjgh-2022-009548 36660904 PMC9445824

[2] Wolff BJ Bramley AM Thurman KA : Improved detection of respiratory pathogens by use of high-quality sputum with TaqMan array card technology. *J. Clin. Microbiol.* 2017;55:110–121. 10.1128/JCM.01805-16 27795345 PMC5228222

[3] Marks F Liu J Soura AB : Pathogens that cause acute febrile illness among children and adolescents in burkina faso, madagascar, and sudan. *Clin. Infect. Dis.* 2021;73:1338–1345. 10.1093/cid/ciab289 33822011 PMC8528393

[4] Moore CC Jacob ST Banura P : Etiology of sepsis in uganda using a quantitative polymerase chain reaction-based TaqMan array card. *Clin. Infect. Dis.* 2019;68:266–272. 10.1093/cid/ciy472 29868873 PMC6321855

[5] Kwambana-Adams BA Liu J Okoi C : Etiology of pediatric meningitis in west africa using molecular methods in the era of conjugate vaccines against pneumococcus, meningococcus, and haemophilus influenzae type b. *Am. J. Trop. Med. Hyg.* 2020;103:696–703. 10.4269/ajtmh.19-0566 32458777 PMC7410464

[6] Liu J Gratz J Amour C : A laboratory-developed TaqMan array card for simultaneous detection of 19 enteropathogens. *J. Clin. Microbiol.* 2013;51:472–480. 10.1128/JCM.02658-12 23175269 PMC3553916

[7] O’Brien KL Baggett HC Brooks WA : Causes of severe pneumonia requiring hospital admission in children without HIV infection from africa and asia: The PERCH multi-country case-control study. *Lancet.* 2019;394:757–779. 10.1016/S0140-6736(19)30721-4 31257127 PMC6727070

[8] Pholwat S Liu J Taniuchi M : Use of molecular methods to detect shigella and infer phenotypic resistance in a shigella treatment study. *J. Clin. Microbiol.* 2022;60:e01774–e01721. 10.1128/JCM.01774-21 34669456 PMC8769730

[9] Liu J Pholwat S Zhang J : Evaluation of molecular serotyping assays for shigella flexneri directly on stool samples. *J. Clin. Microbiol.* 2021;59:10–1128. 10.1128/JCM.02455-20 PMC8111134 33239379

[10] Platts-Mills JA Liu J Rogawski ET : Use of quantitative molecular diagnostic methods to assess the aetiology, burden, and clinical characteristics of diarrhoea in children in low-resource settings: A reanalysis of the MAL-ED cohort study. *Lancet Glob. Health.* 2018;6:e1309–e1318. 10.1016/S2214-109X(18)30349-8 30287127 PMC6227251

[11] Platts-Mills JA Houpt ER Liu J : Etiology and incidence of moderate-to-severe diarrhea in young children in niger. *J. Pediatric Infect. Dis. Soc.* 2021;10:1062–1070. 10.1093/jpids/piab080 34468743 PMC8719619

[12] *Crt, a relative threshold method for qPCR data analysis on the QuantStudio 12K Flex system with OpenArray technology.* Thermo Fisher Scientific; Accessed on August 31, 2024. Reference Source

[13] Burdukiewicz M Spiess A-N Rafacz D : PCRedux: A quantitative PCR machine learning toolkit. *J. Open Source Softw.* 2022;7:4407. 10.21105/joss.04407

[14] Burdukiewicz M Spiess A-N Blagodatskikh KA : Algorithms for automated detection of hook effect-bearing amplification curves. *Biomol. Detect. Quantif.* 2018;16:1–4. 10.1016/j.bdq.2018.08.001 30560061 PMC6287529

[15] Chen T Guestrin C : Xgboost: A scalable tree boosting system. *Proceedings of the 22nd acm sigkdd international conference on knowledge discovery and data mining.* 2016; pp.785–794.

